# Hemp-Straw Composites: Gluing Study and Multi-Physical Characterizations

**DOI:** 10.3390/ma12081199

**Published:** 2019-04-12

**Authors:** Marie Viel, Florence Collet, Sylvie Prétot, Christophe Lanos

**Affiliations:** 1 Laboratoire Génie Civil et Génie Mécanique, Université de Rennes, BP 90422 Rennes, France; florence.collet@univ-rennes1.fr (F.C.); sylvie.pretot@univ-rennes1.fr (S.P.); christophe.lanos@univ-rennes1.fr (C.L.); 2 Institut de recherche en Génie Civil et en Génie Mécanique, Université de Nantes, BP 92208 Nantes, France

**Keywords:** sustainable building materials, hemp shiv, wheat straw, green binders, porosity, thermal properties, mechanical properties

## Abstract

In order to meet the requirement of sustainable development, building materials are increasingly environmentally friendly. They can be partially or fully bio-based or recycled. This paper looks at the development of fully bio-based composites where agro-resources are valued as bio-based aggregates (hemp) and as binding materials (wheat). In a previous work, a feasibility study simultaneously investigated the processing and ratio of wheat straw required to ensure a gluing effect. In this paper, three kinds of hemp-straw composites are selected and compared with a hemp-polysaccharides composite. The gluing effect is analyzed chemically and via SEM. The developed composites were characterized multi-physically. They showed sufficiently high mechanical properties to be used as insulating materials. Furthermore, they showed good thermal performances with a low thermal conductivity (67.9–69.0 mW/(m·K) at 23 ${}^{{}^{\circ}}$C, dry).

## 1. Introduction

This work set out to develop new bio-based building insulating materials from the valuation of locally available agro-resources which are currently minimally or not valued, as part of the European ISOBIO project [1]. Bio-based composites from the use of agricultural co-products also have the advantage of storing CO${}_{2}$ (low carbon footprint) during the utilization phase of their life cycle. Moreover, their hygric and thermal performances have to reduce the energy needs of buildings and ensure better indoor hygrothermal comfort. Firstly, the co-products of crops from flax, hemp, corn, rape, and wheat, produced in France, were characterized chemically and multi-physically in order to identify the possibilities of valuation for the production of building insulating materials. In particular, these characterizations have highlighted that agro-resources have a very interesting chemical composition for use as binders. Indeed, they are mainly composed of polysaccharides [2]. So, the gluing performances of agro-resources were tested with the production of hemp-straw composites. Furthermore, hemp shivs are light aggregates with high porosity (approximately 77% total accessible porosity [3]), and their use is interesting from a thermal point of view. Historically, hemp shivs have been used with mineral binder [4,5] to produce hemp composite with low density and low thermal conductivity. Investigations have also been performed with alternative mineral binders to reduce the environmental impact of the material [6,7,8]. At present, investigations are being performed on agro-based binders [9,10,11].

LigniCell® compressed straw panels are an example of a very interesting bio-based composite. Indeed, no additional components are added to ensure a minimum cohesion between the straws. The wheat straws are just cleaned and compressed between two hot plates in order to realize a hydrothermal treatment at 200 ${}^{{}^{\circ}}$C [12]. The cohesion of the panels is ensured by the lignin (between 8% and 17%), the hemicellulose (between 28% and 33%), and the cellulose (between 33% and 42%) contained in the wheat straw [13]. Moreover, moisture control during the production process ensures a sufficient availability of the water for the reaction. It appears possible to develop a fully bio-based composites by using only agro-resources. Indeed, the components contained in agro-resources could ensure the cohesion of the aggregates to one another if a thermal stress is applied to the composite during the production process.

Following the manufacturing method of compressed straw panels, a preliminary study was carried out to verify the feasibility of a binder made from agro-resources and showed the interesting characteristics of these composites from a hygrothermal point of view. So, composites were made with hemp (used as aggregate) and wheat straw (used as a binder). Their performances were identified to evaluate whether using agro-resources with this production process is interesting for the development of rigid and environmentally responsible insulating panels [14].

This paper focuses on the gluing performances of wheat straw after hydrothermal treatment and the thermal properties of hemp-straw composites. Firstly, the developed materials are presented. Their physical properties (apparent and absolute densities and porosity) and moisture buffer value (MBV) are given. Then, the gluing properties are chemically analyzed (Van Soest analysis), morphologically verified (scanning electron microscopy, SEM) and mechanically tested (compression test) in order to ensure that the composites are usable. Finally, this paper investigates the thermal properties of the developed composites, referring to the objectives in terms of the energy efficiency of buildings.

## 2. Materials and Methods

### 2.1. Developed Materials

In order to develop a fully bio-based composite, this study focused on the use of hemp shiv as aggregate and wheat straw as gluing material. A reference composite was also produced with polysaccharides as binder.

**Figure 1:**
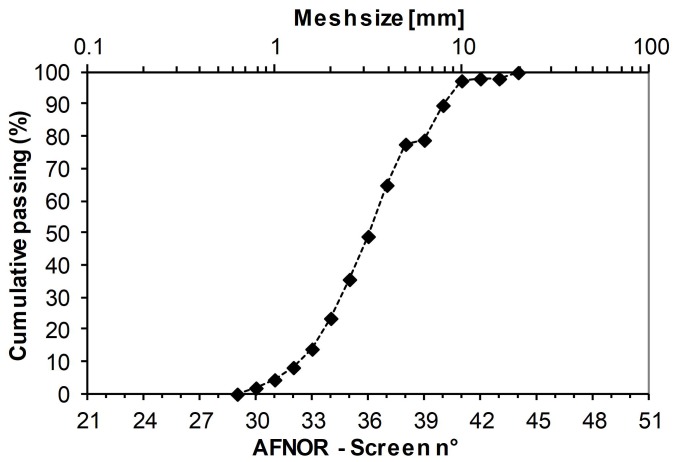
Particle size distribution of hemp shiv (Chanvribat from LCDA–France).

The hemp shiv is a commercial product (Chanvribat from LCDA, Avrillé, France) commonly used to producehemp concrete. More recently, hemp shivs have been used with PLA (polylactic acid) or with starch [5,15,16]. Its bulk density ranges from 100 to 110 kg/m${}^{3}$. The average width of aggregates (W${}_{50}$) is 4 mm, and they have a length-to-width ratio of about 4. The particle size distribution obtained by mechanical sieving is given in Figure 1.

**Figure 2:**
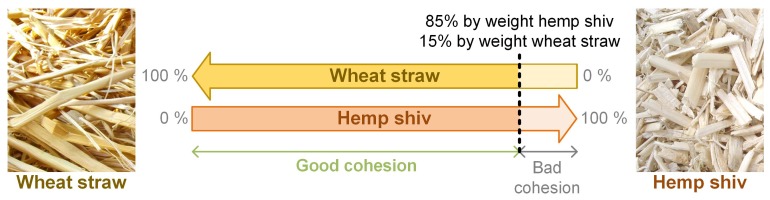
Cohesion versus formulation of hemp shiv-milled wheat straw composites.

For the production of composites, wheat straw (provided by the local agriculture of Acigné, France) was used in different ways. First, the wheat straw was finely chopped, then mixed with hemp. Then, the hemp-straw mix was moistened, with a dry mix-to-water ratio of 1, compressed five successive times at 0.25 MPa, and heated in the mold at 180 ${}^{{}^{\circ}}$C for 100 min. Several hemp shiv-wheat straw ratios were tested. With such processing, a good cohesion is ensured when the wheat straw content in the dry mix is at least 15% by weight (Figure 2). For lower wheat straw content, the specimens were easily breakable by hand. Finally, the selected mix was composed of 80% by weight of hemp shiv and 20% by weight of milled wheat straw (composite A) to ensure high enough mechanical resistance and easy handling.

**Figure 3:**
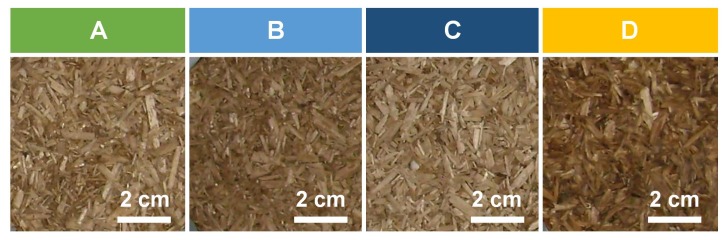
Surface aspect of developed composites.

After checking the good cohesion of the composites, other tests were carried out from the infusion of the wheat straw. It was infused for 24 h in water and heated to 80 ${}^{{}^{\circ}}$C to induce extraction of soluble components. In a first step, the mix of the infusion with wheat straw and hemp shiv was processed as previously described (composite B). In a second step, the infused wheat straw was filtered and only the extracts in solution were mixed with hemp shiv. The mix was then processed as previously described (composite C). This process allows the quantity of wheat straw to be reduced in order to ensure the gluing effect. Finally, after the optimization of the production process, four developed composites were selected: three hemp-straw composites with different wheat straw processing and one hemp-polysaccharide composite (Table 1). For each formulation, three specimens (numbered i, ii, and iii) of 10 cm diameter and 7 cm height (Figure 3) were produced.These three specimens were characterized in the following order: thermal conductivity measurement at (23 ${}^{{}^{\circ}}$C, dry), compression test, SEM, and pycnometry.

The composites were light, their density ranged from 166 to 188 kg/m${}^{3}$ at 23 ${}^{{}^{\circ}}$C; 50%RH (Table 2). Their skeleton density was about 1500 kg/m${}^{3}$, leading to very high total porosity of about 89%. For hemp-straw composites, the composite with the highest hemp content showed the lowest density and the highest porosity (C vs. A and B).

In terms of moisture, the ability of the materials to moderate the variations of indoor humidity in buildings is characterized by their moisture buffer value (MBV). The MBV of the developed composites was investigated in a previous study [14]. The average MBV of the developed composites was around 2.27 g/(m${}^{2}$·%RH) (±3%) even though the MBV were similar with the three composites glued with straw, the MBV was slightly higher for the composite made with polysaccharides [14]. Thus, the MBV was not impacted by the density of the composite while it seemed to be slightly impacted by the binder. According the Nordtest classification [17], all these composites are thus excellent hygric regulators (MBV > 2 g/(m${}^{2}$·%RH)).

Hemp–straw composites had MBVs in the high range of values found in the bibliography. For hemp–lime composites, the MBVs range from 1.94 to 2.24 g/(m${}^{2}$·%RH) [15,18], while for hemp-PLA, the MBVs are approximately 1.77 g/(m${}^{2}$·%RH) [15].

### 2.2. Characterization Methods

#### 2.2.1. Van Soest Method

The evolution of the chemical composition of wheat straw was studied along the course of processing: without treatment, after infusion at 80 ${}^{{}^{\circ}}$C (hydrothermal treatment 1), and then after infusion at 80 ${}^{{}^{\circ}}$C and heating at 180 ${}^{{}^{\circ}}$C (hydrothermal treatment 2).

**Figure 4:**
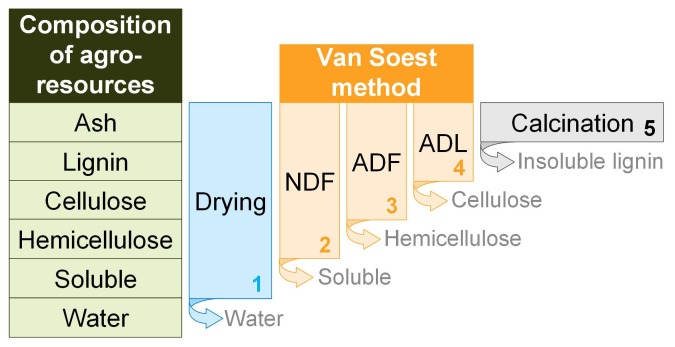
Synthetic sketch of the Van Soest method allowing assay of the biomass composition [20]. ADF: acid detergent fiber; ADL: acid detergent lignin; NDF: neutral detergent fiber.

The Van Soest method [19,20,21,22] consists of successive extractions to identify the composition of agro-resources (Figure 4).

The agro-resources are crushed, sieved through a 1-mm mesh, and poured into a porous bag. Then, the sample is exposed to the action of neutral, acidic detergents, and concentrated sulphuric acid in order to solubilize the solubles, hemicellulose, and cellulose in succession. Weight loss is used to determine the content of the various components present in agro-resources. Finally, the content of lignin is determined following a calcination. The method is given in more detail in Viel et al. [2].

#### 2.2.2. Surface Morphology by Scanning Electron Microscopy

SEM was used to view the impact of the treatment on the aggregates and the gluing between aggregates and binder. The aggregates were glued with carbon tape whereas the composite samples were glued with Araldite glue. Then, the specimens (aggregates and composites) were coated with a layer of palladium (thickness of about 30 nm) before the characterization. Scanning electron microscopy (SEM) was performed with a JSM 7100F (JEOL, Tokyo, Japan) equipped with an Everhart-Thornley secondary electron detector and Schottky field emission.

#### 2.2.3. Mechanical Characterization

Compressive tests were performed with a Zwick/Roell ProLine testing machine (Ulm, Germany) fitted with a 20 kN XForce load cell (load up to 0.02% of its full capacity and 0.05% readability). The tests were carried out in displacement with a cross-head speed equal to 0.05 mm·s${}^{-1}$. The loading was monotonic (no loading cycles). The samples were placed between two steel plates in order to guarantee a homogeneous displacement and pressure. The load was applied by the displacement of the upper plate. The test was performed on three cylindrical samples of 10 cm diameter and 7 cm height for each formulation.

The results of the mechanical tests were analyzed using stress–strain curves, according to the NF EN 826 standard [23]. The stress was assessed by reporting the load to the initial surface of the sample, and the deformation was relative to the initial height of the the sample. The origin of the stress–strain curve was adjusted in order to prevent the contact effects between the plates and the surface of the samples, which was not perfectly flat.

#### 2.2.4. Thermal Characterization

The measurement of thermal conductivity was performed with a transient method: hot wire, following the method described by Collet and Prétot [24]. The measurement was realized with a commercial “*CT Meter*” device (SMEE, Voiron, France) equipped with a five-centimeter-long hot wire. The power was 142 mW and the heating time was 120 s.

**Figure 5:**
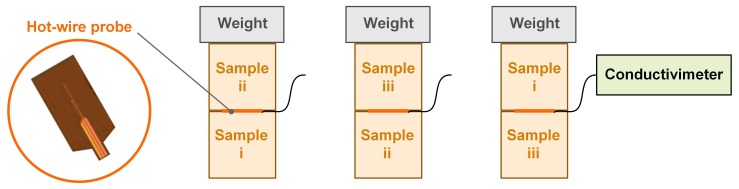
Measurement of thermal conductivity: Schematics of hot wire probe (**left**) and specimen coupling (**right**).

Prior to the measurements, the cylindrical specimens of 10 cm diameter and 7 cm height were dried at 60 ${}^{{}^{\circ}}$C in an oven. Then, the measurements were performed after weight stabilization at 23 ${}^{{}^{\circ}}$C at dry state in a desiccator and after weight stabilization at 23 ${}^{{}^{\circ}}$C, 50%RH in a climate chamber. For each formulation, three pairs of specimens (i&ii, i&iii, and ii&iii) were measured. The thermal conductivity of a pair was considered to be the average of three values with a coefficient of variation lower than 5%. The thermal conductivity of a composite was considered to be the average of the values obtained for the three pairs (Figure 5).

## 3. Results

### 3.1. Binding Component: Treated Wheat Straw

#### 3.1.1. Chemical Composition

**Figure 6:**
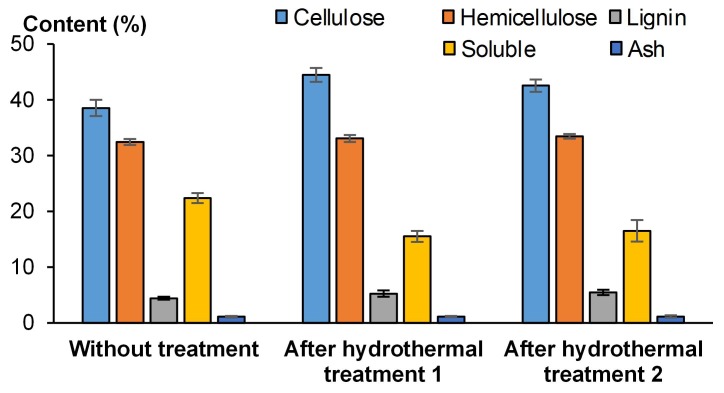
Chemical composition of wheat straw without treatment and after hydrothermal treatments 1 and 2.

The hydrothermal treatment induced a weight loss in the wheat straw, which increased with the treatment temperature. After treatment and drying, the weight loss was 3.46% after hydrothermal treatment 1 (infusion at 80 ${}^{{}^{\circ}}$C) and it was 5.86% after hydrothermal treatment 2 (infusion at 80 ${}^{{}^{\circ}}$C and heating at 180 ${}^{{}^{\circ}}$C). Table 3 and Figure 6 give the relative chemical composition of wheat straw without treatment, and after hydrothermal treatments 1 and 2. The hemicellulose and ash contents remained unchanged regardless of the treatment. However, the relative cellulose and lignin contents increased by 15.25% and by 18.74% after the infusion, respectively, while the soluble content decreased by 30.56%. These differences can be explained by the weight loss after the treatments. Indeed, the water-soluble components were removed during the treatments and most of those components were a low fraction of lignin, ash, and hemicellulose and a larger fraction of pectins, which are included in solubles. On the other hand, the heat depolymerized water-soluble and insoluble components and released acid molecules which initiated repolymerization reactions [25,26,27]. These polymers may play the role of binder in the composites.

These results were compared with literature values. Rajput et al. [28] also hydrothermally treated wheat straw at different temperatures. They noted an increase in cellulose, and a decrease in hemicellulose, lignin, and other components with increasing temperature. Ran et al. [29] hydrothermally treated washed vinegar residue and found an increase in cellulose, a slight increase in lignin, and a decrease in hemicellulose with increasing temperature. These results differ between each other and from what was observed in this paper. Thus, the chemical composition of wheat straw depends on its origin, including the effects of the area of production, the weather (i.e., sunlight, relative humidity, temperature, rainfall, and wind), and the variety and maturity of the plant [2]. Moreover, agro-resources do not always react in the same way during hydrothermal treatment because the hemicellulose, lignin, and pectins are composed of different monomer units which are not connected and distributed in the same way. Thus, the polymers have different properties.

#### 3.1.2. Surface Morphology by Scanning Electron Microscopy

**Figure 7:**
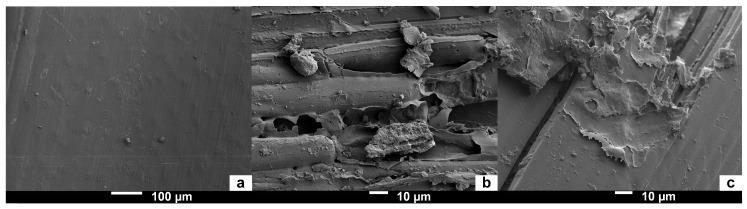
SEM micrographs of the wheat straw surface: (**a**) Without treatment; (**b**,**c**) After hydrothermal treatment 2.

Figure 7 presents SEM micrographs of the surface of wheat straw, before and after hydrothermal treatment 2. The infusion allows the removal of soluble components such as lignin, hemicellulose, pectins, waxes, and fat from the cell wall. Thus, these components can easily react with each other in liquid medium under the effect of heat. Indeed, in the SEM micrographs in Figure 7a, the external surface of the wheat straw is perfectly smooth apart from the presence of a few starch granules, whereas the external surface was slightly damaged after hydrothermal treatment 2, as shown by the SEM micrographs in Figure 7b,c.

### 3.2. Developed Composites

#### 3.2.1. Surface Morphology by Scanning Electron Microscopy

**Figure 8:**
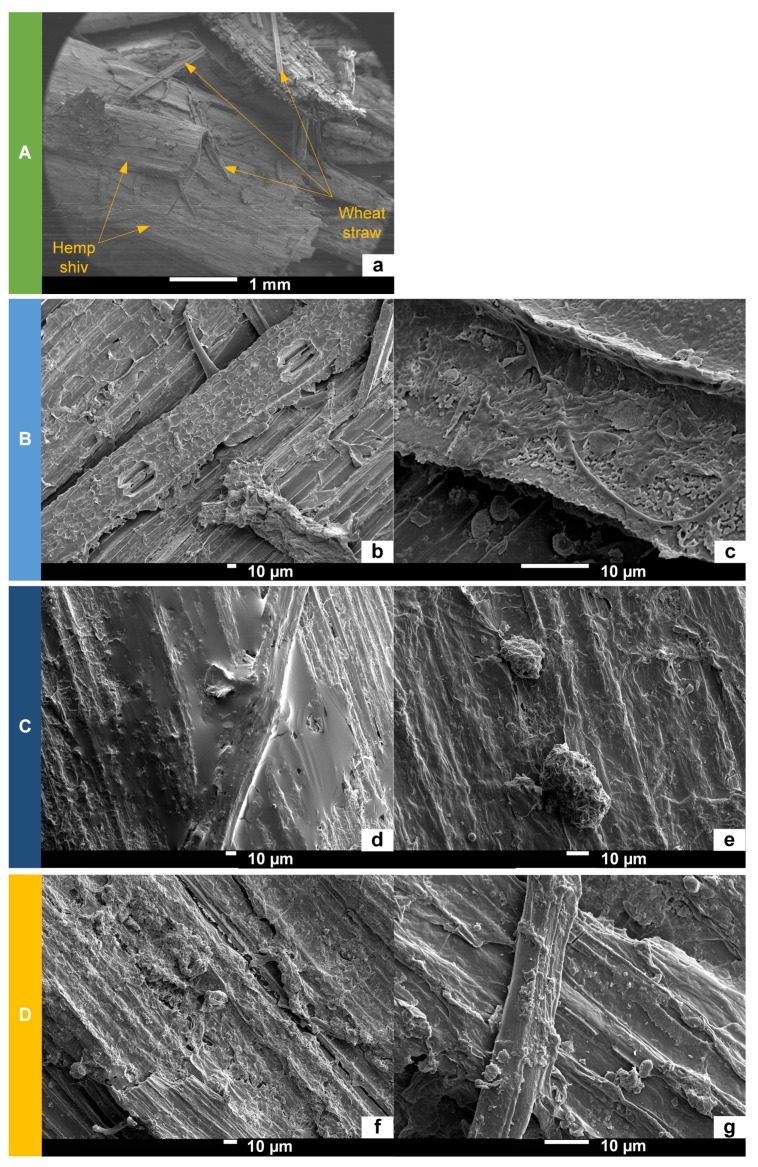
SEM micrographs at the interface between the hemp shiv and the binder: (**a**) composite A, (**b**,**c**) composite B, (**d**,**e**) composite C, (**f**,**g**) composite D.

Figure 8 presents SEM micrographs at the interface between the hemp shiv and the binder. For all composites, SEM analysis indicated good adhesion at the interface, showing several hemp shivs well-coated and glued together. There were microstructural differences at the interface between the hemp shiv and the different binders.

The surface of composite A could not be zoomed further because the sample was charged under the electron beam. However, the SEM micrograph in Figure 8a shows hemp shiv and wheat straw glued together. It is supposed that the surface aspect was similar to composite B, but likely with the hemp shiv less thoroughly coated. Indeed, the two composites contained wheat straw in their formulation, but the wheat straw was not previously infused in composite A.

The surface of composite B (Figure 8b,c) was rough and well coated by the binder.

The surface of composite C (Figure 8d,e) had a similar roughness to composite B. However, a fragile smooth film surrounded a small hemp shiv but several fracture zones were visible above. Some binder granules of 10–20 μm in diameter were also visible on the composite surface. Thus, the binder distribution was less homogeneous than for composite B.

The surface of composite D (Figure 8f,g) also had a similar roughness to composite B, even if the density of the binder was lower than for composite B. Besides, some binder granules with diameter of 2–20 μm were also visible on the composite surface, as for composite C. Thus, the binder distribution was less homogeneous than for composite B and more homogeneous than for composite C.

Therefore, the wheat straw (milled or infused and/or extracts) seemed to have a similar good gluing performance to polysaccharides.

#### 3.2.2. Mechanical Characterization

**Figure 9:**
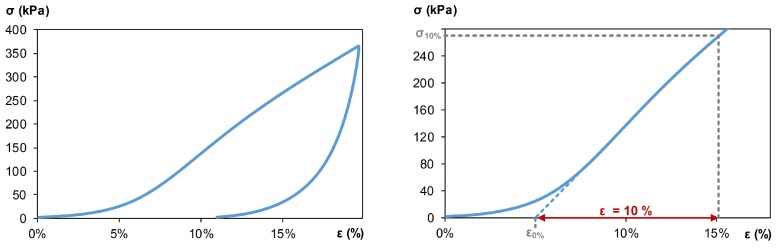
Stress-strain curves for composite A.i.

**Figure 10:**
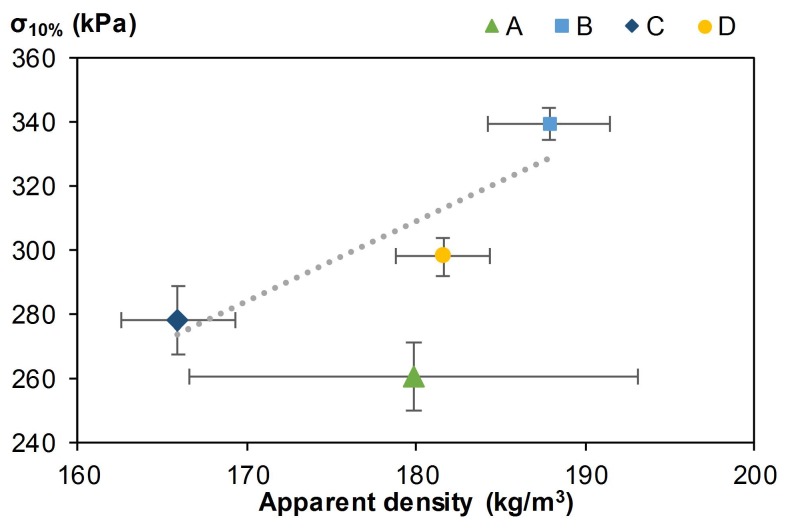
Stress at 10% deformation versus apparent density at 23 ${}^{{}^{\circ}}$C and 50%RH of composites.

The developed composites showed compacting behavior (Figure 9). So, the mechanical performance was given by the compressive strength obtained for longitudinal strain ϵ = 10% [23]. The mechanical properties of composites are presented in Figure 10 and Table 4. Compressive strength varied between 260 and 339 kPa for the four composites. Composite B had the highest compression strength and composite A the lowest. As shown in Figure 10, the compressive strength increased with apparent density and crosslinking density [30], which were induced by the quantity of binder, in composites B, C, and D. Composite A did not fit with this curve because the wheat straw was not previously infused. Thus, it was more difficult to have a homogeneous crosslinking density since the solubles that allow gluing were not in solution. Apparent elastic modulus could be estimated from the stress-strain slope at 10% deformation. Values ranged from 2.6 to 3.4 MPa.

The compressive strength at 10% deformation of all composites was higher than 250 kPa. For stress induced by density corresponding to walls of 3 m height, the obtained deformations ($\epsilon_{h=3m}$) were lower than 0.20%. So, the mechanical resistance and rigidity were sufficient for use as self-bearing insulation panels.

Compared with compressive strength at 10% deformation obtained in the literature, these values were lower. Indeed, Balčiūnas et al. [31], who studied the hemp-sapropel (sediments that are rich in organic matter) composites, obtained better results as they ranged from 360 to 2080 kPa for density ranging from 220 to 410 kg/m${}^{3}$. Additionally, the hemp-starch composites developed by Bourdot et al. [11] had slightly better mechanical properties. The compressive strength at 25% deformation ranged from 570 to 630 kPa for density ranging from 182 to 188 kg/m${}^{3}$, whereas for the same deformation, composites A, B, C, and D had a compressive strength of around 500 kPa.

#### 3.2.3. Thermal Characterization

**Figure 11:**
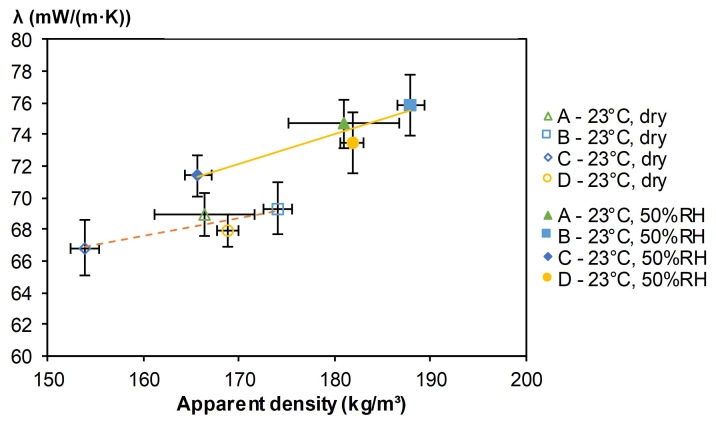
Thermal conductivity of composites at (23 ${}^{{}^{\circ}}$C, dry) and at (23 ${}^{{}^{\circ}}$C, 50%RH).

Table 5 and Figure 11 give the thermal conductivity of hemp-straw and hemp-polysaccharides composites. In all cases, the correlation coefficient between experimental data and fitting curve was very high (>0.9997). Moreover, a great confidence in thermal conductivity measurements was made possible by a low coefficient of variation (<3% for the nine measurements).

The thermal conductivities of developed composites ranged from 66.8 to 69.3 mW/(m·K) at 23 ${}^{{}^{\circ}}$C, dry state and from 71.4 to 75.9 mW/(m·K) at 23 ${}^{{}^{\circ}}$C, 50%RH. Regardless of the kind of composite (glued with straw or with polysaccharides), the thermal conductivity increased with the density in the same trend (Figure 11). So, the method of gluing hemp shiv does not seem to have much impact on thermal conductivity. The thermal conductivity increased by about 8% from dry state to wet state at 23 ${}^{{}^{\circ}}$C, 50%RH for all composites.

The thermal conductivities of developed composites were lower than the values found in our literature review for hemp-lime composites, mainly thanks to density. Actually, in Collet and Prétot [24], the thermal conductivities ranged from 93 to 120 mW/(m·K) at 23 ${}^{{}^{\circ}}$C, 50%RH for respective densities between 260 and 390 kg/m${}^{3}$. De Bruijn and Johansson [32] found thermal conductivity values ranging from 100 to 116 mW/(m·K) at 65%RH for two lime-hemp mixes when the densities were between 298.1 and 394.8 kg/m${}^{3}$ respectively. For composites made with hemp shiv and PLA, the thermal conductivity ranged from 85 mW/(m.K) at 260 kg/m${}^{3}$ to 120 mW/(m·K) at 350 kg/m${}^{3}$ [15].

Moreover, the thermal conductivity of hemp-straw composites were also close to the thermal conductivity obtained on hemp-starch composites by Tran Le [16] (62 mW/(m·K) for a density of 176 kg/m${}^{3}$ at dry state). Thus, the density of hemp composites is the main factor influencing the thermal conductivity.

Finally, the thermal conductivities of the developed composites were close to the value needed to be considered as insulating building material (65 mW/(m·K)) [33]. As the thermal conductivity decreased with density, one way to reach this value involves reducing the density of composite. This can be done by reducing compaction during processing, without changing binder content.

## 4. Conclusions

This paper highlights the feasibility of using wheat straw as a binder to produce 100% bio-based rigid insulating composites. This target was reached thanks to the valuation of local agricultural wastes: hemp shiv as aggregate and wheat straw as binder.

The minimum quantity required to ensure good cohesion was 15%–20% wheat straw in the dry mix (and 80%–85% hemp shiv). Indeed, the soluble components of the wheat straw played the role of binder after the hydrothermal treatment, which initiated their depolymerization and repolymerization reactions. They were mainly composed of pectins, lignin, and hemicellulose. Firstly, these components were solubilized in water at 80 ${}^{{}^{\circ}}$C. Secondly, the polysaccharides degraded, condensed, then recombined during the hydrothermal treatment at 180 ${}^{{}^{\circ}}$C.

SEM analysis indicated the good gluing performances of wheat straw on the developed composites because there was a good adhesion between the aggregates (hemp shiv) and the binder (treated wheat straw). Indeed, the SEM micrographs highlight that hemp shivs were well coated and glued together.

The apparent density of developed composites was quite low, ranging from 165 to 190 kg/m${}^{3}$, depending on the processing method. Their mechanical performances were sufficient for use as rigid insulating panels. Moreover, their mechanical performances increased with apparent density and crosslinking density, which are induced by the quantity of binder in the case of similar binders. The thermal conductivity of hemp-straw composites ranged from 67.9 to 69.0 mW/(m·K) at 23 ${}^{{}^{\circ}}$C, dry. They were quite low and close to the value needed to be considered as building insulating material [33]. Furthermore, the developed composites were excellent hygric regulators, with very close MBV values (higher than 2.20 g/(m${}^{2}$·%RH)) [14].The main factor influencing the multiphysical properties of the composites was the density. Indeed, the composite with the lowest bulk density (composite C) also had the best thermal conductivity, whereas the composite with the highest bulk density (composite B) also had the best mechanical performance.

Thus, these results are encouraging. Hemp-straw composites allow the objectives of the ISOBIO project to be met, as they are fully bio-based and they show thermal conductivity and hygric performances which contribute to reducing the energy needs of building projects and to ensure hygrothermal comfort to users. As thermal conductivity increases with density, one way to improve it could consist of reducing density. This could be reached by reducing compaction during processing.

## Figures and Tables

**Table 1:** Formulation of composites: component mass (g) and binder processing (wheat straw or polysaccharides).

Composites	A	B	C	D
Hemp shiv	192	192	192	190
Wheat straw	48	48	30	-
*Processing*	*Milled*	*Infused and Extracts*	*Extracts*	*-*
Polysaccharides	-	-	-	20
Water	240	360	320	200

**Table 2:** Physical characteristics of composites: apparent density at 23 ${}^{{}^{\circ}}$C, 50%RH, and 23 ${}^{{}^{\circ}}$C, dry state, skeleton density, porosity, and moisture buffer value (MBV) in absorption, desorption, and average [14].

Composites	A	B	C	D
$\rho_{23^{{}^{\circ}}C-50\%RH}$ (kg/m${}^{3}$)	179.8 ± 13.2	187.9 ± 3.6	165.9 ± 3.4	181.6 ± 2.8
$\rho_{23^{{}^{\circ}}C-dry}$ (kg/m${}^{3}$)	166.5 ± 12.1	174.1 ± 3.3	153.9 ± 3.4	168.8 ± 2.7
$\rho_{s}$ (kg/m${}^{3}$)	1529.4 ± 6.0	1509.8 ± 16.9	1496.7 ± 21.2	1475.8 ± 8.0
$n_{tot}$	89.1%	88.5%	89.7%	88.6%
MBV${}_{abs}$ (g/(m${}^{2}$·%RH))	2.23 ± 0.02	2.17 ± 0.03	2.21 ± 0.02	2.36 ± 0.01
MBV${}_{des}$ (g/(m${}^{2}$·%RH))	2.30 ± 0.02	2.23 ± 0.02	2.24 ± 0.01	2.47 ± 0.01
MBV${}_{av}$ (g/(m${}^{2}$·%RH))	2.27 ± 0.02	2.20 ± 0.03	2.22 ± 0.02	2.42 ± 0.01

**Table 3:** Chemical composition of wheat straw without treatment and after hydrothermal treatments 1 and 2 (1: infusion at 80 ${}^{{}^{\circ}}$C, 2: infusion at 80 ${}^{{}^{\circ}}$C and heating at 180 ${}^{{}^{\circ}}$C).

Agro-Resources	Cellulose (%)	Hemicellulose (%)	Lignin (%)	Soluble (%)	Ash (%)
Without treatment	38.56 ± 1.47	32.45 ± 0.54	4.43 ± 0.26	22.38 ± 0.89	1.11 ± 0.15
Hydrothermal treatment 1	44.44 ± 1.26	33.11 ± 0.62	5.26 ± 0.55	15.54 ± 0.98	1.14 ± 0.12
Hydrothermal treatment 2	44.52 ± 1.07	33.45 ± 0.42	5.49 ± 0.48	16.51 ± 1.94	1.16 ± 0.22

**Table 4:** Stress at 10% deformation for each composite.

Composites	A	B	C	D
$\rho_{23^{{}^{\circ}}C-50\%RH}$ (kg/m${}^{3}$)	179.84 ± 13.22	187.85 ± 3.63	165.92 ± 3.40	181.57 ± 2.81
$\sigma_{10\%}$ (kPa)	260.72 ± 10.49	339.07 ± 5.35	276.62 ± 12.71	298.05 ± 5.99
$\epsilon_{h=3m}$ (%)	0.19	0.15	0.16	0.16

**Table 5:** Thermal conductivity of composites (mW/(m·K)) versus density at (23 ${}^{{}^{\circ}}$C, 50%RH) and at (23 ${}^{{}^{\circ}}$C, dry).

Composites	A	B	C	D
$\rho_{23^{{}^{\circ}}C-dry}$ (kg/m${}^{3}$)	166.5 ± 5.2	174.1 ± 1.4	153.9 ± 1.5	168.8 ± 1.2
$\lambda_{23^{{}^{\circ}}C-dry}$ (mW/(m·K))	69.0 ± 1.3	69.3 ± 1.6	66.8 ± 1.8	67.9 ± 1.0
$\rho_{23^{{}^{\circ}}C-50\%RH}$ (kg/m${}^{3}$)	181.1 ± 5.8	188.0 ± 1.4	165.7 ± 1.4	181.9 ± 1.3
$\lambda_{23^{{}^{\circ}}C-50\%RH}$ (mW/(m·K))	74.7 ± 1.6	75.9 ± 1.9	71.4 ± 1.3	73.5 ± 1.9

## Abbreviations

The following abbreviations are used in this manuscript:

MBV	Moisture Buffer Value
SEM	Scanning Electron Microscopy
